# Quantitative genetic analysis of life-history traits of *Caenorhabditis elegans *in stressful environments

**DOI:** 10.1186/1471-2148-8-15

**Published:** 2008-01-22

**Authors:** Simon C Harvey, Alison Shorto, Mark E Viney

**Affiliations:** 1 School of Biological Sciences, University of Bristol, Woodland Road, Bristol, BS8 1UG, UK

## Abstract

**Background:**

Organisms live in environments that vary. For life-history traits that vary across environments, fitness will be maximised when the phenotype is appropriately matched to the environmental conditions. For the free-living nematode *Caenorhabditis elegans*, we have investigated how two major life-history traits, (i) the development of environmentally resistant dauer larvae and (ii) reproduction, respond to environmental stress (high population density and low food availability), and how these traits vary between lines and the genetic basis of this variation.

**Results:**

We found that lines of *C. elegans *vary in their phenotypic plasticity of dauer larva development, *i.e*. there is variation in the likelihood of developing into a dauer larva for the same environmental change. There was also variation in how lifetime fecundity and the rate of reproduction changed under conditions of environmental stress. These traits were related, such that lines that are highly plastic for dauer larva development also maintain a high population growth rate when stressed. We identified quantitative trait loci (QTL) on two chromosomes that control the dauer larva development and population size phenotypes. The QTLs affecting the dauer larva development and population size phenotypes on chromosome II are closely linked, but are genetically separable. This chromosome II QTL controlling dauer larva development does not encompass any loci previously identified to control dauer larva development. This chromosome II region contains many predicted 7-transmembrane receptors. Such proteins are often involved in information transduction, which is clearly relevant to the control of dauer larva development.

**Conclusion:**

*C. elegans *alters both its larval development and adult reproductive strategy in response to environmental stress. Together the phenotypic and genotypic data suggest that these two major life-history traits are co-ordinated responses to environmental stress and that they are, at least in part, controlled by the same genomic regions.

## Background

Organisms live in environments that vary both spatially and temporally. In such variable environments there are different ways to maximise fitness. Life-history traits can either be robust to environmental change (a buffered or canalised trait) or they can be variable in an environmentally-dependent manner (a phenotypically plastic trait). Phenotypic plasticity of a trait can be manifest as a continuous phenotypic range across an environmental gradient, such as the variation in *Drosophila melanogaster *body size metrics across temperature ranges [1]. Alternatively, phenotypic plasticity may appear as a threshold trait, with apparently distinct phenotypes developing in different environments. An example of this is the switch between winged and wingless aphid morphs in response to host plant quality and, or aphid population density [2]. These different phenotypic responses have been termed phenotypic modulation and developmental conversion, respectively [3].

*A priori*, fitness could be maximised by all traits being fully phenotypically plastic. However, phenotypically plastic traits vary both within and between populations, particularly in the magnitude and sensitivity of their response to environmental change: in the language of phenotypic plasticity, there may be different reaction norms. The existence of this variation suggests that there are limits or costs to the evolution of phenotypically plastic traits and of the reaction norms of traits, and therefore that fitness is maximised by not all traits being fully phenotypically plastic. These costs are likely to be (i) having sufficiently accurate and robust processes for environmental sensation, (ii) maintaining the genetic and cellular machinery for the development of alternate phenotypes and (iii) co-ordination between different phenotypically plastic traits [4-6]. Therefore, all traits can be considered on a continuum from those that are fully plastic, via those with a low level plasticity, to non-plastic, invariant traits. The molecular basis of the phenotypic plasticity of most traits is not clear, but progress in identifying genes involved in such environmental interactions is being made (e.g [7-10]). For many organisms, including intensively studied 'model' species, the role and function of the majority of genes remains unknown [11,12]. It is probable that genes involved in phenotypically plastic traits, especially the means by which the phenotype is modulated in response to the environment, are among these genes with, as yet, unidentified functions. Given this, it is crucial to move towards integrating an understanding of the molecular basis of phenotypic plasticity with the ecology of the species in question [13].

The model free-living nematode *Caenorhabditis elegans *has a phenotypically plastic developmental switch in its life-cycle. In the 'normal' *C. elegans *life-cycle, progeny moult through four larval stages (L1 – L4) into adult worms. Under conditions that are unsuitable for growth and reproduction an alternative, developmentally arrested third larval stage, the dauer larva, is formed [14,15]. Dauer larvae are environmentally resistant, have a greatly extended lifespan compared with non-dauer larvae, and will only resume normal development as fourth stage larvae (L4) when exposed to more favourable conditions. This developmental conversion (dauer *vs*. non-dauer) is made based on the worms' perception of environmental quality, which is determined by the concentrations of both food and dauer pheromone and temperature. Dauer pheromone is a cue produced by all worms that acts as a measure of con-specific population density [16] and appears to consist of three related molecules [17,18]. The relative concentrations of food and dauer pheromone are used to assess environmental quality: dauer larvae develop under low food and high dauer pheromone concentration conditions, *i.e*. conditions of environmental stress; 'normal', non-dauer, larvae develop under high food and low dauer pheromone concentration conditions, *i.e*. conditions of plenty.

Geographically distinct isolates of *C. elegans *vary extensively in the phenotypic plasticity of dauer larva formation [19]. That is, for the same change in environmental conditions, there is variation in the proportion of larvae that develop into dauer larvae. The adaptive value of these different plasticities of dauer larva formation is not known. There has been extensive investigation into the genetic and molecular genetic control of the development of dauer larvae [14,15]. However, the genetic basis of the variation between isolates in the phenotypic plasticity of dauer larva development is not known. Furthermore, the fitness consequences of different phenotypic plasticities of dauer larva development are not known.

In the *C. elegans *life-cycle there are other phenotypic responses to conditions of environmental stress. For example adult lifespan, lifetime fecundity, the schedule of reproduction and body size all vary as a consequence of food quantity and quality [20,21]. At present little is known about variation between *C. elegans *isolates in these genotype by environment (GxE) interactions and it is not known if they represent separate trait-specific responses to environmental change or are part of a co-ordinated response. Hence, it is possible that the fitness of a *C. elegans *genotype may be maximised by a co-ordinated response to environmental stress of both larval and adult traits. Further, it can be envisaged that different strategies may have evolved for how such major larval and adult life-history traits co-ordinately respond to environmental change.

To investigate this, we have analysed recombinant inbred lines (RILs) produced from *C. elegans *isolates N2 and DR1350, which differ significantly in their plasticity of dauer larvae formation [19]. Using these, we determined how the development of dauer larvae and the population growth, as assayed by determining population size through time, varied between the RILs. Population size at any given time is a consequence of both survival and all the factors that contribute to reproduction (*e.g*. time to first reproduction, total lifetime fecundity, rate of reproduction) this broad analysis was most likely to identify any relationship with dauer larvae development. We found that the *C. elegans *RILs vary in both their phenotypic plasticity of dauer larva development and their population size and that these traits are related. Specifically, we found that lines of *C. elegans *that are highly plastic for dauer larva development also maintain a high population growth rate when stressed (*i.e*. their rate of reproduction is comparatively unchanged under conditions of environmental stress). This suggests that *C. elegans *may co-ordinate its larval development and adult reproductive strategy to respond to environmental stress. We have also mapped the quantitative trait loci (QTL) that control the phenotypic plasticity of dauer larva development and of population size. These analyses have identified QTL that affect dauer larva development and population size.

## Results

### *C. elegans *varies in its phenotypic plasticity of dauer larva formation

**Figure 1:**
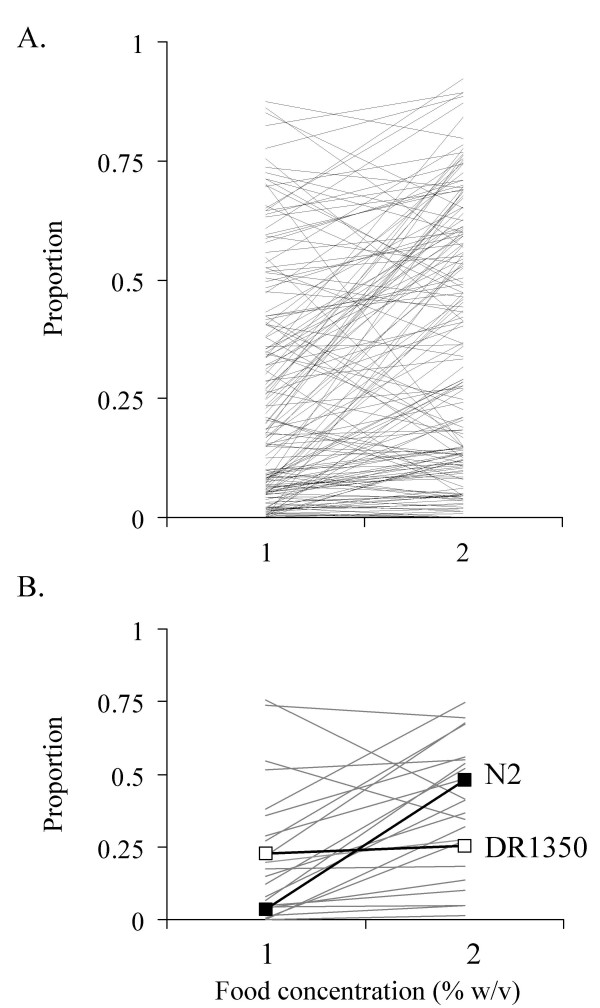
***C. elegans *varies in its dauer larvae formation phenotype**. (A) The proportion of dauer larvae that developed at two concentrations of food (1 and 2% w/v *E. coli *OP50 in water) for 163 RILs and (B) this for 21 RILs and N2 (-■-) and DR1350 (-□-) in one representative assay.

The proportion of dauer larvae that developed in 163 RILs at two different (1% and 2% w/v) food concentrations, *i.e*. the reaction norm of dauer larvae development, is shown in Figure 1A; for clarity these data from one representative assay are shown in Figure 1B. RILs that are highly plastic for dauer larva formation have the greatest positive difference in the proportion of dauer larvae formed at low food concentrations, compared with high food concentrations (*i.e*. the steepest, positive, reaction norms); N2 is an example of such a line (Figure 1B). Other lines are less plastic; DR1350 is an example of such a line (Figure 1B). Approximately a quarter of the RILs (45/163) had a negatively plastic dauer larva development phenotype (*i.e*. a reaction norm with a negative slope); that is, a lower proportion of dauer larvae developed at a low food concentration, compared with a high food concentration (Figure 1A). In many of these 45 lines, the difference in dauer larva formation between the two food concentrations was very small and, given the inherent variation in this trait and its assay, it is likely that these lines have only a small, or no, plasticity of dauer larva development. However, ten lines had a negative plasticity ≥ 0.2, and were found to consistently show this response in additional assays (data not shown).

As expected dauer larva formation was significantly affected by food concentration (FOOD: F_1,542 _= 4.64, *p *= 0.048). There was significant variation between the RILs (LINE [ASSAY]: F_153,542 _= 6.43, *p *< 0.001) and the RILs also varied significantly in their dauer larva formation phenotype in response to changes in the concentration of food (FOOD*LINE [ASSAY]: F_153,542 _= 1.55, *p *< 0.001), *i.e*. there is genetic variation between the RILs for both overall propensity to form dauer larvae and in their plasticity of dauer larva development with respect to food concentration. There were significant differences between the assays, and in the effect of food concentration between the assays (ASSAY: F_9,542 _= 6.43, *p *< 0.001; FOOD*ASSAY: F_9,542 _= 3.28, *p *< 0.001).

The dauer larva development phenotype with respect to dauer pheromone concentration had previously been determined for 35 of these RILs [19]. We used these data to compare the dauer larva development of these lines with respect to changes in the concentration of food and of pheromone. This showed that there was a significant positive correlation between these plasticities of dauer larvae development (r^2 ^= 0.40, *p *= 0.02). This, therefore, suggests that the plasticity of dauer larva development is, at least partially, independent of the particular environmental cue (*i.e*. food or pheromone concentration) used for the initiation of dauer larva development.

### *C. elegans *varies in its population growth

**Figure 2:**
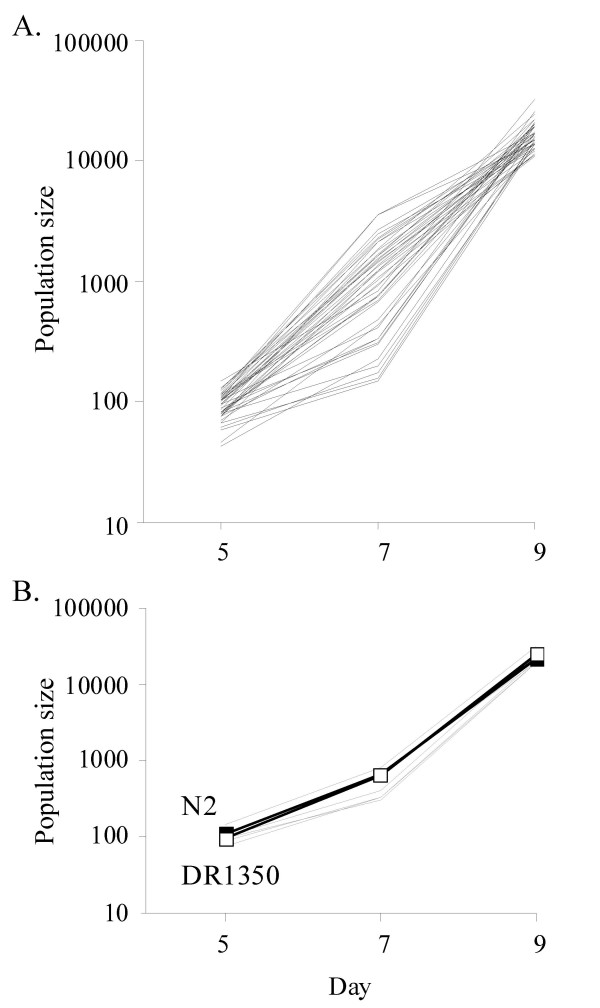
***C. elegans *varies in its population growth rate**. (A) The population size on days 5, 7 and 9 of 45 RILs and (B) this for six RILs and N2 (-■-) and DR1350 (-□-) in one representative assay.

We wished to determine how *C. elegans *varied for another important life-history trait, namely adult reproduction. To do this we determined the population size of 45 RILs as they grew. This showed that the population sizes of the lines increased over time, but that the rate of this increase differed between the lines (Figure 2A; for clarity these data from one representative assay are shown in Figure 2B). There were significant differences between the population sizes of the lines and in how they grew over time (TIME: F_2,1039 _= 23431.01, *p *= 0.048; LINE [ASSAY]: F_37,1039 _= 9.98, *p *< 0.001; TIME*LINE [ASSAY]: F_74,1039 _= 4.14, *p *< 0.001). There were also significant differences between the assays, and in how the populations grew between assays (ASSAY: F_7,1039 _= 130.84, *p *< 0.001; TIME*ASSAY: F_14,1039 _= 102.49, *p *< 0.001). Hence, the RILs vary in their population sizes and in how the populations grew over time. At the start of these assays (day 5) there are no limitations to population growth; as the size of the population increases (day 7), food conditions become limiting and by day 9, the food source is exhausted. We therefore posited that the variation between the RILs was due to both differences in the reproductive capacity of the lines (the effect of LINE), possibly manifest either by variation in their lifetime fecundities or their temporal schedule of reproduction, and by differences in how the reproductive capacity of the lines altered in response to density (the effect of TIME*LINE [ASSAY]). These possible explanations were investigated further.

### *C. elegans *varies in its fecundity

One of the principal features of high density environments is a reduction in the *per capita *food availability. To investigate how the fecundity of *C. elegans *was affected by this we firstly determined the lifetime fecundity of 12 RILs under *ad libitum *food conditions. This showed that these RILs had significantly different lifetime fecundities (LINE [BLOCK]: F_10,222 _= 10.35, *p *< 0.001); fecundity did not differ between experimental blocks (BLOCK: F_1,222 _= 3.14, *p *= 0.078). We calculated the rate of reproduction, *q*, a measure of the rate of increase in the proportion of lifetime fecundity, of the RILs and found that this also differed significantly between RILs (LINE [BLOCK]: F_10,222 _= 7.94, *p *< 0.001) and between experimental blocks (BLOCK: F_1,222 _= 131.46, *p *< 0.001).

These data were used to calculate the expected population size of the lines in the absence of any limits on population growth on days 5, 7 and 9. In all cases, the population size was greater than that which was observed. These predicted population sizes correlated with the observed population size on day 7 (r^2 ^= 0.548, *p *= 0.002), but not on days 5 and 9 (r^2 ^= 0.097, *p *= 0.26 and r^2 ^= 0.12, *p *= 0.207, respectively). This may suggest that at day 7, growing populations of *C. elegans *are not constrained by density-dependent effects, but at day 9 that they are. The absence of a significant correlation on day 5 may be due to inter-RIL variation in the rate of reproduction.

We next considered how lifetime fecundity and the rate of reproduction, and hence the expected population sizes of the lines, was affected by low concentrations of food, a likely feature of high-density populations. Analogously, as above, the lifetime fecundity differed between the lines (LINE: F_5,223 _= 10.85, *p *< 0.001) and was significantly negatively affected by food concentration (FOOD: F_3,223 _= 35.63, *p *< 0.001). But, the lines did not differ in how their lifetime fecundities were affected by different food concentrations (LINE*FOOD: F_15,223 _= 1.30, *p *= 0.20). The rate of reproduction, *q*, did not differ between lines (LINE: F_5,223 _= 1.33, *p *= 0.25), but it was significantly affected by the food concentration (FOOD: F_3,223 _= 8.80, *p *< 0.001), such that at lower food concentrations *q *was lower. The lines also differed significantly in how *q *was affected by food concentration (FOOD*LINE: F_15,223 _= 2.41, *p *= 0.003). Together, these results therefore show that the rate of reproduction was affected by food concentration independently of effects on lifetime fecundity.

### Why does low food concentration reduce fecundity?

The fecundity of wild-type *C. elegans *is reduced when there is limited food available. We sought to investigate whether this occurs due to (i) direct effects of caloric restriction *per se *or (ii) indirect effects on reproduction due to the perception of food availability, *i.e*. altered resource allocation in response to perceived food limitation. To do this we compared reproduction under a range of food concentrations in mutants defective in pharyngeal pumping with reproduction in wild-type worms. This was done with strains with mild pharyngeal defects (DA573 *eat-14(ad573) *and DA602 *eat-15(ad602)*), with severe pharyngeal defects (DA522 *eat-13(ad522) *and DA606 *eat-10(ad606)*) [21] and N2 as a control. Here, as far as it is known, the perception of food by all strains will be the same, but the actual acquisition of food will be compromised in the strains with pharyngeal defects, and more strongly so in those with the more severe defects. *i.e*. the comparison allowed the separation of the acquisition of food from the perception of food. We hypothesised that if caloric restriction (i, above) controlled the food concentration-dependent change in the rate of reproduction, *q*, the rank order of the food-dependent reduction in *q *would be *eat-10 *and *eat-13*; *eat-14 *and *eat-15*; N2. In contrast, if food perception (ii, above) controlled the food concentration-dependent reduction in the rate of reproduction, *q*, then no relationship between strength of the pharyngeal defects and food-dependent reduction in fecundity would be observed.

The mutants with the strongest pharyngeal defects (*eat-10*, *eat-13*) had the lowest rate of reproduction, *q*, and this was reduced most under lower food concentrations, compared with mutants with mild pharyngeal defects (*eat-14 *and *eat-15*) and compared with N2 (Table 1). This therefore suggests that the food concentration-dependent reduction in the fecundity of *C. elegans *is directly due to caloric restriction.

**Table 1:** Rates of reproduction at different food concentrations

		Food concentration (% w/v)				
		
Pharyngeal defect	Line	100	50	25	12.5	6.25
**None**	**N2**	0.56 ± 0.02	0.50 ± 0.01	0.51 ± 0.02	0.45 ± 0.01	0.34 ± 0.01
**Mild**	***eat-14***	0.48 ± 0.01 (0.86)	0.47 ± 0.01 (0.94)	0.45 ± 0.01 (0.88)	0.38 ± 0.01 (0.84)	0.31 ± 0.03 (0.91)
	***eat-15***	0.45 ± 0.03 (0.80)	0.49 ± 0.01 (0.98)	0.49 ± 0.02 (0.96)	0.47 ± 0.02 (1.04)	0.37 ± 0.04 (1.09)

**Strong**	***eat-10***	0.42 ± 0.01 (0.75)	0.42 ± 0.01 (0.84)	0.40 ± 0.01 (0.78)	0.34 ± 0.01 (0.75)	0.25 ± 0.01 (0.74)
	***eat-13***	0.31 ± 0.01 (0.55)	0.30 ± 0.01 (0.6)	0.30 ± 0.01 (0.59)	0.26 ± 0.01 (0.58)	0.23 ± 0.01 (0.68)

The rates of reproduction, *q*, (± 1 SE) for N2 and four *eat *mutants at a range of food concentrations. Values in parentheses are the proportionate change of the rate of reproduction compared to N2 at the same food concentration.

### QTL analysis

#### Polymorphic markers

To identify markers that would differentiate N2 and DR1350, 144 genomic regions of DR1350 were sequenced and a further 49 candidate polymorphisms were screened by restriction fragment length polymorphism (RFLP) analysis (Table 2 and additional file 1). Overall, 45 polymorphisms were detected that differentiated N2 and DR1350; this included 20 polymorphisms previously identified [22]. 42 of these 45 markers were used to genotype the RILs (the remaining three markers were developed at a later date and used only in the fine-mapping, see below). These markers cover chromosomes I, II, III and X (Table 2). In total, 15.45 Kbp of chromosome IV and 12.60 Kbp of chromosome V of DR1350 were sequenced, but no polymorphisms between N2 and DR1350 were detected (see additional file 1 for details).

**Table 2:** Polymorphic markers differentiating N2 and DR1350

		Name	N2/DR1350	Location	Forward	Reverse	Enzyme
**I**	1	pkP1003	G/A	126950	gtatcctcatccttctaccacc	gcgtcgttccacgtgttatgc	*Rsa*I
	2	pkP1098	C/T	360847	tatcatgctggcgtagatttc	tggataaaaagcgtttctgg	*Hpa*II
	3	pkP1051	A/T	825028	cctacaacaggcaaagaagc	aattcctaccaaagctccgc	*Ssp*I
	4	pkP1102	C/A	1517890	tggaaggatattgtggcg	ctgaacgcgattctcctgtg	*Hinf*I
	5	pkP1016	T/G	1882334	gacaatgaccaataagacg	gatccgtgaaattgttccg	*Bsr*I
	6	snp_F28H1[1]	C/T	3989631	tgccaaaatagcagtaggc	tgaaactgcaataacataacg	*Hpy*CH4V

**II**	1	W08F4/33109	T/C	593962	cagacttccaccgtaccattg	gagacgaaacgatttacgagg	*Dra*I
	2	pkP2134	C/T	862482	atcaggatccggaacagtcg	tcgtaaatggtcagttttgg	*Dde*I
	3	pkP2010	T/C	1129821	taatttctagcaccagtgaggc	cccaaatttccacctgtaatcc	*Dde*I
	4	pkP2015	A/G	1640462	gtacctaccgtcattgatagtg	ctttcagtggacagaatccg	*Pvu*II
	5	pkP2136	C/T	1683953	agttgtgttatacttgttgg	tgtctaactgaagagatgacg	*Xba*I
	6	T05A8/28596	G/A	2631509	ctatggtgcatcgaagtgtc	gtcagcacgttcttaaccttg	*Bam*HI
	7	pkP2026	T/C	2737466	cgatggattatgtggtgagtc	caggttggtcatcatttcagac	*Sty*I
	8	pkP2114	C/G	2755074	tcacgtcgtcacctacgcc	aatctgaccaaggtatcgg	*Alu*I
	9	nP133	-/TTCCG	2769667–8	aacgttcaaaagtgataggtc	ttctcactcggtgtactcgg	*Eco*RI
	10*	snp_Y25C1A[1]	C/A	3077209	accgtctttcagcgctcgacg	caaaattctgctgataatgg	*HpyCH*4V
	11	F19B10/12159	G/T	3660181	ctgcttctctgcaagtctgc	cttggaacagtctctcaacg	*Dra*II
	12*	snp_C49D10[3]	A/T	3868386	agaacatctatcacgacttgg	tttgtgtcatatttgcgtcc	*Tsp509*I
	13	pkP2051	G/A	4299265	gcgtgttttttccgtcgatcg	cagcgtccagactggtttgg	*Hae*II
	14*	snp_B0304[5]	GG/TT	4526832–3	acatcaccaattacacgacc	agatcgtacttattgtagcc	*Eco*0109I
	15	C18A3/8661	G/A	5710053	catgtggacgacgtgtactgg	caatgtgcagtcgtctactgg	*Pvu*I
	16	K10B2/5930	C/T	6362593	ccttgtactcgggaacacgc	gaatcagtcaaacgctgcgg	*Sfu*I
	17	pkP2109	C/T	9401077	tgaacccataacagcttctgc	aactcgtgcgctctccttgg	*Eco*RI
	18	pkP2069	G/T	10489659	tcaaccttcatacgtgtcgc	ggaatgactgataaaggtgtcg	*Xba*I
	19	snp_W01G7[1]	G/C	14044928	tatataggtacctaaagagc	atttttgtccccttatatgg	*Cac*8I

**III**	1	pkP3002	A/T	743326	ctgcttatagtcttcctgtcg	gcaaccccaccttcaatgac	*Ssp*I
	2	snp_Y46E12[4]	T/C	1765120	tctaatgttttttccaatcagc	tatgattttactgctgctgg	*Ssp*I
	3	pkP3099	T/C	5625446	tctttcagtgggctaacacc	tgcgtgggcagcccaaatacg	*Hpy*CH4V
	4	B0361/15143	A/C	7279588	gtatttcttacccgagagtcc	cagttcacctggaatctcaatc	*Dde*I
	5	snp_C50C3[2]	G/A	8180784	gctcttcttggtacgttccc	cgcgtcttctgagtgtttcc	*Alu*I
	6	snp_T05G5[1]	T/A	9742847	cgtaaactaccaaactcggtg	ggtctactacaactatacaggc	*Dra*I
	7	pkP3059	A/G	10613168	actcggccacgtggcaagc	aaagcctttcggaacttcc	*Xba*I
	8	snp_F56A8[1]	C/T	13247630	tttggaggaccatcagagg	tggctcaccttctttctcc	*Sau*3A

**X**	1	snp_T23F2[1]	T/A	5492084	tttccggcagatgcaccacg	tgcaaatagctgatcactgg	*Dde*I
	2^1^	C03B1/38853–6	TATA/CG	6374380–3	cgtagagacgcaaaataggc	gctcaatttcacgcgtccag	*Acc*I
	3^1^	C25B8/5416	C/G	6374374	gtatctgagatagggtgcgc	cggaaaacctgttagacatgg	*Xba*I
	4^1^	F41C6/10186	A/G	6879298	gtttggtcgctggagttttgg	catcaaagaggcaacaagggg	*Fsi*I
	5^2^	pkP6158	C/T	8691678	aagacacccatccatgcatatc	caattgtcagccgttgtttc	*Hind*III
	6^2^	pkP6030	T/G	8814093	gtaatcggttactgtgcactg	ctacatcaatgtcaacaccagc	*Dde*I
	7	pkP6137	C/T	9677614	gccttggagagtctcgatttg	ttctgaccaccatagccgaac	*Hinf*I
	8	pkP6039	T/C	10652947	cctcatctcatctttgcttg	caagatgacttgccgattcatg	*Mse*I
	9	snp_F23D12[1]	T/C	14429597	taaacagaaaaattcacaaa t/c	atatttgaatggagtttcacc	†
	10	pkP6167	C/T	15106709	taatagccgccaaagtgcg	gtgaagcaagtttgattttcc	*Hinf*I
	11	snp_C02C6[7]	A/G	15561287	ttcgcgcatttatcttgtcc	tatattcattgatctaagtgc	*Hpy*CH4V
	12	pkP6096	A/T	16389174	gattgaacatagctcacagc	tttcgatcgttttggacgcc	*Rsa*I

For chromosomes I, II, III and X, for each polymorphism, its name, the N2/DR1350 polymorphism, its physical location in N2 [23], the forward and reverse PCR oligonucleotide primers (5'-3') and, where appropriate, the restriction enzyme used for genotyping. * denotes markers only used in the analysis of the NILs. 1 and 2 denote two groups of markers that could not be separated genetically. † denotes the marker scored in the RILs by allele specific PCR.

188 RILs were genotyped at 42 loci. Ten of these RILs were found to have an N2 genotype at all loci and these were excluded from all analyses. For the remaining 178 RILs, the measured genetic distance between markers was greater than the F2-derived genetic maps [23], with a map expansion of 3.45, 2.73, 2.43 and 3.21 for chromosomes I, II, III and X, respectively. On the X chromosome a group of three markers (2, 3 and 4) and a pair of markers (5 and 6) (Table 2) could not be separated genetically and so the non-informative markers were removed from all further analyses. Analysis of the 39 remaining markers revealed extensive segregation distortion among the RILs, with the overall allele frequency of the 39 markers in the 178 RILs biased to N2 (65%) and a variable rate of segregation distortion across chromosomes. This implies that there was selection for the N2 alleles at many of the loci during the construction of the RILs, a finding common in many studies analysing *C. elegans *RILs (e.g. [9,24,25]).

#### Dauer larva development QTLs

**Figure 3:**
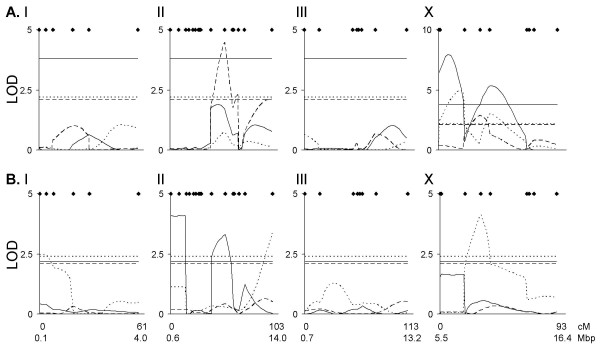
**QTL analysis of dauer larva formation and population size**. LOD scores for chromosomes I, II, III and X for QTL mapping of (A) dauer larva formation in response to high (—) and low (- - -) food concentrations and the plasticity of dauer larva formation (· · ·) and the respective genome-wide LOD significance thresholds for these traits, some of which are co-incident, shown as horizontal lines and (B) the population size on days 5 (—), 7 (- - -) and 9 (· · ·) and the respective genome-wide LOD significance thresholds for these traits, shown as horizontal lines. For each chromosome, the genetic distance between the outermost markers and their physical location is given [23]. Note that in (A), the LOD scale differs for the X chromosome. Positions of the markers described in Table 2 are shown by ◆.

Comparative interval mapping (CIM) of dauer larvae formation in response to different food concentrations in 153 RILs identified six QTLs: two each for the plasticity of dauer larva formation, and for dauer larva formation at high and low food concentrations (Figure 3, Table 3). Five of these QTL occurred on the X chromosome, one, (for dauer larva formation at low food concentration) occurred on chromosome II. Multiple trait CIM (MT-CIM) analysis of the dauer larva formation at high (2% w/v) and at low (1% w/v) food concentrations supported the QTLs identified by CIM for dauer formation at low food concentration and one of the QTLs for dauer formation at high food concentration (marker 2 on the X chromosome from Table 3). Similarly, one of the QTLs identified by CIM for the plasticity of dauer larvae formation (marker 2 on the X chromosome from Table 3) was supported the MT-CIM analysis (significant GxE interaction detected in that area of the X chromosome). CIM and MT-CIM analyses of dauer larvae formation in response to different pheromone concentrations in 35 RILs identified no significant QTLs. However, there were significant single marker effects predominantly on the X chromosome (Table 3).

**Table 3:** Summary of QTL mapping

		Population size			Dauer [food]			Dauer [pheromone]		
				
		Day 5	Day 7	Day 9	H	L	P	L	H	P
			
**I**	1									
	2									
	3									
	4				*	*		*		
	5							*		
	6									
			
**II**	1	*								
	2	0.34 (-19)				*				
	3			*						
	4	*								
	5									
	6									
	7					*				
	8					*				
	9					*				
	11				*					
	13	0.2 (18)			***	0.07 (-0.1)				
	15				*					
	16				*	*				
	17				*	*				
	18			0.33 (-3050)						
	19					*				*
			
**III**	1									
	2									
	3					*				
	4					**				
	5					*				
	6									
	7				**	**				
	8				***	**				*
			
**X**	1				***		*			
	2	*		*	0.28 (0.15)		0.17 (-0.12)			*
	5					0.07 (-0.08)	**			*
	7			0.22 (-2612)	0.15 (-0.14)	***			*	*
	8				**	**	0.12 (0.1)			*
	9				*					*
	10				**					*
	11				*					
	12									*

Summary of the genetic mapping by single marker and CIM of population size on days 5, 7 and 9, dauer larva formation for high (H) and low (L) concentrations of food or pheromone and the respective plasticity (P) of dauer larva formation. For the CIM, the estimated locations and effects of QTL significant at a genome-wide level are shown as the proportion of the inter-RIL variance explained by the QTL, R^2^, and, in parentheses, the magnitude of the effect of the QTL where a positive value indicates that the DR1350 allele increases the trait value compared to the N2 allele. Marker-trait associations not identified in the CIM analysis, but which where significant by single marker analysis are indicated by asterisks, with *, ** and *** denoting significance at the 5%, 1% and 0.1% levels, respectively.

**Figure 4:**
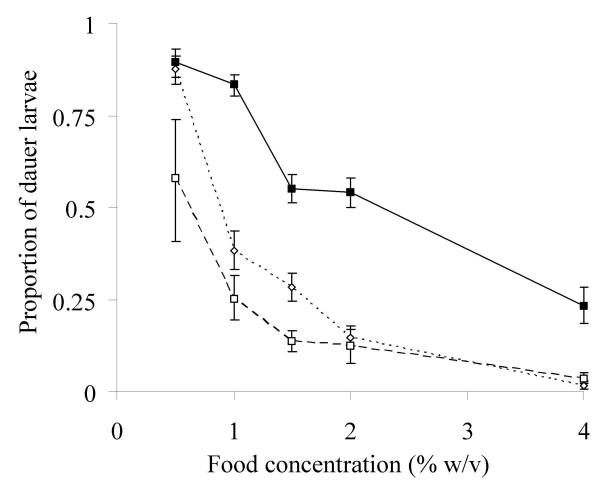
**A chromosome II QTL affects dauer larva development**. The mean (± 1 SE) proportion of dauer larvae that developed over a range of food concentrations for N2 (-■-), DR1350 (-□-) and NIL-1 (···◇···).

The chromosome II QTL affecting dauer larva formation at low food concentration had only a relatively small phenotypic effect (Table 3). However, analysis of dauer development in nearly isogenic lines (NILs), in which the chromosome II QTL region of the DR1350 genome was introgressed into N2, suggests that this QTL has a much greater effect and that it affects the dauer larva formation phenotype across a range of food concentrations. This is shown most clearly in the comparison of NIL-1 to N2 and DR1350 over a range of food concentrations (Figure 4).

#### Population size QTLs

CIM analysis of the population size at 5, 7 and 9 in 35 RILs identified two QTL each for population size on day 5 and on day 9, three of which were on chromosome II and one on chromosome X (Figure 3, Table 3). MT-CIM analysis of population size supported one of the day 5 population size QTLs on chromosome II (marker 2 on chromosome 2 from Table 3). No significant QTLs were detected by CIM for population size on day 7 nor were any significant single marker associations identified.

Combined, the analyses of dauer development and population size have therefore identified two regions of the *C. elegans *genome that affect both traits. Firstly, there is a region of the X chromosome near marker 2 that significantly affects both the population size on day 9 and dauer larva formation in response to changes in both the food and pheromone concentrations, by CIM and single marker analyses respectively. Comparison of these phenotypes among the 21 RILs for which both phenotypes had been measured showed that the population size on day 9 was positively correlated with the plasticity of dauer larva formation in response to pheromone concentration (r^2 ^= 0.55, *p *= 0.01), but not with the plasticity in response to food concentration (r^2 ^= 0.10, *p *= 0.57). Secondly, there is a region on chromosome II near marker 11 (see below) that significantly affects both the population size on day 5 and dauer larva formation at low food concentrations. However, these traits do not correlate (r^2 ^= 0.10, *p *= 0.57).

We undertook further mapping to investigate the extent of the co-incidence of the chromosome II QTLs affecting both population size and dauer larva formation.

### Dauer larva development: chromosome II further mapping

**Figure 5:**
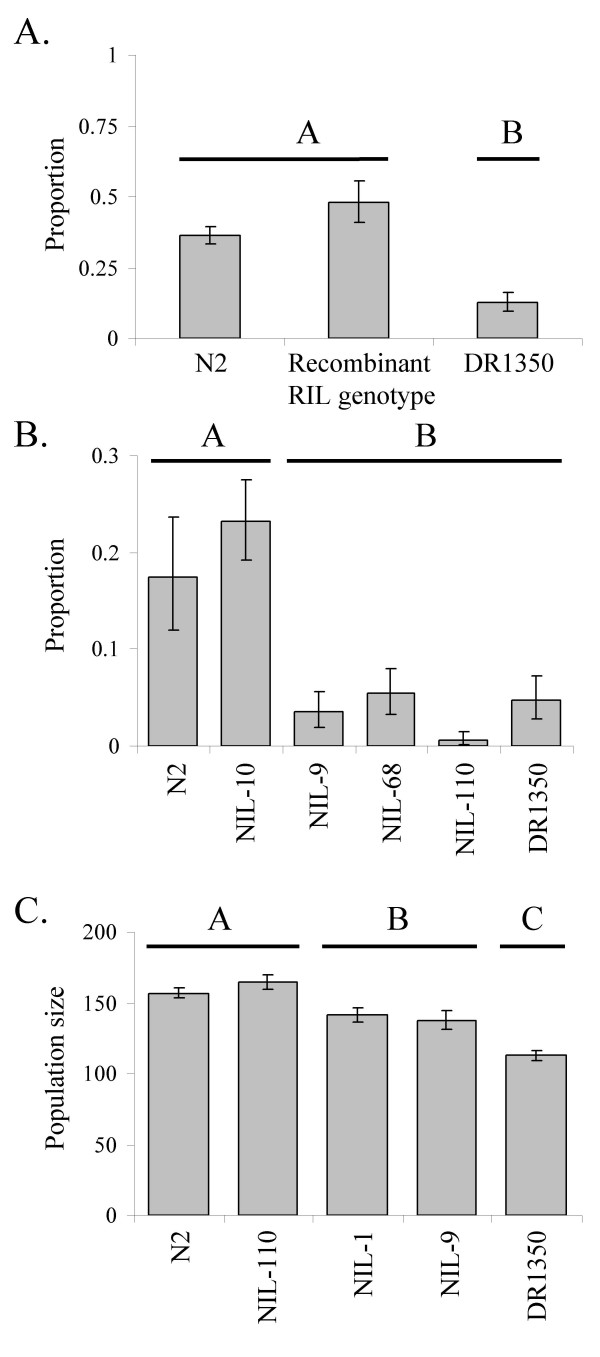
**Dauer larva development and population size: chromosome II further mapping**. The mean (± 1 SE) proportion of dauer larvae that developed at a low food concentration (1% w/v) in (A) RILs with a DR1350, N2 or a N2/DR1350 recombinant genotype for markers 11 and 13 on chromosome II and (B)in N2, DR1350 and four NILs with varying regions of the DR1350 genome introgressed into an N2 background. (C) The mean (± 1 SE) population size on day 5 in N2, DR1350 and three NILs with varying regions of the DR1350 genome introgressed into an N2 background. Within each figure, lines or groups marked with the same letter do not significantly differ; those marked with different letters do significantly differ (p < 0.05).

**Figure 6:**
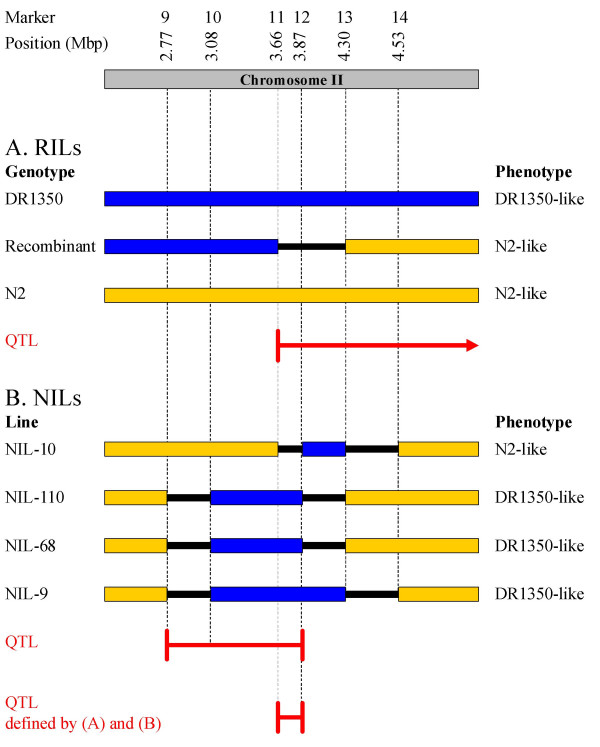
**Schematic representation of the chromosome II QTL**. Schematic representation of a region of chromosome II showing the dauer larva development phenotype of (A) RILs with an N2-like, DR1350-like or recombinant genotype between markers 11 and 13 (Figure 5A), this defines the QTL to lie to the right of marker 11 (red arrow) and (B) N2, DR1350 and four NILs with varying regions of the DR1350 genome introgressed into an N2 background (Figure 5B), which therefore defines the QTL to lie between markers 9 and 12 (red line). The data from A and B combined therefore define the QTL to a 208 Kbp region between markers 11 and 12. DR1350 genotype is shown in blue and N2 genotype in yellow, thinner black lines denote regions where recombination has occurred between flanking markers and the genotype is therefore not known.

Among the RILs, those with a DR1350 genotype at chromosome II markers 11 and 13 (*i.e*. RILs with a DR1350 genotype at the chromosome II QTL) formed a significantly lower proportion of dauer larvae compared with those lines either with an N2 genotype at both markers and recombinants (*i.e*. RILs with a DR1350 genotype at marker 11 and an N2 genotype at marker 13) (F_2,160 _= 11.25, *p *< 0.001) (Figure 5A). This therefore indicates that the loci underlying the chromosome II QTL are not located between markers 9 and 11 and lie to the right of marker 11 on chromosome II (Figure 6A).

Analysis of dauer larva formation in NILs indicated that at a low food concentration (1% w/v), a significantly lower proportion of dauer larvae developed in NILs-9, -68 and -110 and DR1350 than in NIL-10 and N2 (F_5,87 _= 8.42, *p *< 0.001) (Figure 5B), *i.e*. the presence of the introgressed DR1350 DNA is sufficient to replicate the DR1350 dauer development phenotype. The genotype data for these NILs therefore defines the DR1350 allele of the chromosome II QTL affecting dauer development to lie between markers 9 and 12 on chromosome II (Figure 6B). This combined with the previous definition of the left extremity of the QTL (Figure 5A) therefore resolves the QTL to a 208,662 bp region between markers 11 and 12 (3.66 – 3.87 Mbp) (Figure 6).

This chromosome II QTL region in N2 is predicted to contain 72 genes and 10 pseudogenes (see additional file 2A for details) [23]. The RNAi phenotypes, gene ontology (GO) terms, and both the spatial and temporal patterns of expression of these genes do not present any obvious candidates for the control of the dauer larva development phenotype. However, thirteen of these 72 predicted genes and 7 of the 10 predicted pseudogenes are predicted to be 7-transmembrane receptors [23]. The role of these receptors in information transduction, may suggest that a polymorphism in one of these genes or pseudogenes, such that an N2 pseudogene is active in DR1350, may underlie the differences in dauer larva development of these lines.

The 5 QTL identified on the X chromosome could not be defined to small genomic regions. The combined confidence limits for these QTL encompass approximately a third of the X chromosome (5.5 – 12.3 Mbp). Within this region there are a number of genes known to be involved in dauer larva development, *e.g*. *daf-9*, *daf-12*, *dyf-6 *and *dyf-12 *[11,23]. The phenotypic effect of the X chromosome QTL may therefore be due to one of these genes and, or other loci within this region.

### Population size: chromosome II further mapping

Analysis of the population size trait indicated that on day 5, the population sizes of N2 and NIL-110 were significantly greater than those of NILs-1 and -9, which were in turn greater than that of DR1350 (F_4,96 _= 17.86, *p *< 0.001) (Figure 5C). The genotype data for these NILs therefore resolves the DR1350 allele affecting population size on day 5 to a 657,795 bp region between markers 12 and 14 (3.87 – 4.53 Mbp). In N2, this region is predicted to contain 116 genes and 2 pseudogenes (see additional file 2B for details) [23]. The RNAi phenotypes, GO terms and both the spatial and temporal patterns of expression of these genes do not suggest any obvious candidates for the control of the population size phenotype.

## Discussion

Extensive variation in both the phenotypic plasticity of dauer larva development and population size phenotypes among the RILs was observed. For both phenotypes, there were RILs with phenotypes more extreme than the parental lines (Figures 1 and 2). Indeed, such transgressive segregation appears to be common in *C. elegans *and has been observed for a number of other life-history traits (e.g. [9,24,25]). The variation in dauer larva development between RILs was similar in magnitude to that previously observed among a small number of RILs [19]. However, we observed that approximately 5% of the lines had substantial negative plasticity of dauer larva formation; that is, dauer larva formation was greatest at the higher food concentration. A similar phenomenon was observed in the phenotypic analysis of synthesised daumone, where across a range of food concentrations, negative phenotypic plasticity of dauer larva formation was observed at very low food concentrations, but beyond a mid-concentration of food, there was positive phenotypic plasticity [17]. We hypothesise that in the RILs studied here this previously reported negative plasticity phenotype [17] has become manifest for some the RILs because of a shift in these lines of their sensitivity to the concentration of food.

The adaptive value of the *C. elegans *dauer larva and the variation in its formation is unknown, largely due to our limited knowledge of the natural history of this species. In compost-rich soils, *C. elegans *is mostly found as dauer larvae rather than as reproducing adults [26]. The comparative abundance of this life-cycle morph in the wild therefore suggests its central importance for *C. elegans *and that the control of the formation of dauer larvae is likely to be under strong natural selection. This suggests that variation in the plasticity of dauer larva formation may also be under similarly strong selection and hence that this variation may be adaptive.

The observed population sizes of the RILs were smaller than those predicted from measures of lifetime fecundity and reproductive schedule. In these assays on day 9 the food supply is exhausted which suggests that by this time the growing population is subject to density-dependent effects. The *C. elegans *rate of reproduction is lower in low food concentration settings. Comparison of the rate of reproduction of *eat *mutants suggests that the rate of reproduction is determined directly by calorific restriction, thus, that density-dependent effects on reproduction come about by such caloric restriction. This trait is therefore different from the dauer larva developmental choice, in which a developmental decision is made based primarily on the multi-factor perceived environmental conditions. That is, the density-dependent effects on reproduction are a consequence of the internal state of the worm while the effects on dauer development are a consequence of the external environment.

In summary, under conditions of reduced food availability, a likely significant environmental stress, larval stages of *C. elegans *form dauer larvae and the reproduction of the adult hermaphrodites is altered, which reduces the rate of reproduction. Therefore, these different life-cycle stages both have strategies for responding to this environmental stress and that these larval and adult hermaphrodite responses to this stress vary between lines. There are consistent differences in *C. elegans *life-history traits when grown on two different food sources. On chemically defined liquid media, both development and lifetime fecundity are reduced and lifespan increased compared with worms grown on an NGM/OP50 food source [27-29]. These results suggest the existence of at least two distinct adult life histories: one maximizing the intrinsic rate of population increase (NGM/OP50 food), and the other the efficiency of exploitation of the carrying capacity of the environment (chemically defined liquid media) [29]. Our observations that *C. elegans *varies in its reproductive response to altered food conditions, is therefore in general concordance with these observations.

We detected substantial genetic polymorphism between N2 and DR1350 for much of the genome. However, we did not detect any genetic differences between these isolates on chromosomes IV and V nor in ~5 Mbp of the X chromosome. This supports previous observations [22] that also did not detect any polymorphisms between N2 and DR1350 in several regions of the genome, including those noted here.

Our genetic mapping analyses identified 10 QTL that affect either dauer larva development or population size (Figure 3 and Table 3). There are two principal regions of the genome, on chromosomes II and X, that affect both dauer larva development and population size. The dauer larva development and population size QTLs identified on the X chromosome could not be separated and, as would be expected given the position and effects of the day 9 population size and dauer development QTLs on the X chromosome (Table 3), these traits are positively correlated in the RILs. However, given the limited resolution of these QTLs little can be inferred about the genes underlying these QTLs. In contrast, detailed analysis of the chromosome II region separates the day 5 population size and dauer larva development phenotypes (Figure 5) showing that they are not controlled by the same loci. Crucially, the chromosome II region that can control dauer larva formation does not include any genes previously identified to affect this phenotype; therefore a new locus or loci that affects dauer larva development has been discovered. This locus or loci may not have been identified previously by more usual mutagenesis analyses, perhaps because there may not be a severe (or any) dauer formation (*daf*) loss of function phenotype.

The QTLs for population size appeared, on average, to have greater phenotypic effects compared with those affecting dauer larva development (Table 3). However, for the chromosome II dauer larvae development QTL, the phenotypic effect size is greater in the NIL analysis compared with the QTL analysis (Figure 4 and 5C) and this QTL affects dauer development across a range of food concentrations (Figure 4). The difference between the effect size observed in the RILs and the NILs could have a number of explanations. It is unlikely that there are epistatic interactions between the DR1350 introgressed region and the N2 genome, since no such interactions were detected between QTLs. Similarly, we consider it unlikely that the difference between the estimated phenotypic effect size of the QTL in the RILs and the observed effect size in the NILs is due to the separation in the NILs of loci with opposing effects on dauer larva formation given the relatively large size (10.0 – 11.3 Mbp of chromosome II, data not shown) of the DR1350 region introgressed in NIL-1. The greater than expected phenotypic effect of the QTL in the NILs may therefore be an underestimate due to variation in trait measurement in these analyses (Figure 1). Alternatively, the increased effect of the QTL in the NILs may be a consequence of the use of daumone, rather than pheromone extract in the assay of the NILs. Because daumone is only one of three active compounds in dauer pheromone [18], a larger observed effect would be expected in the NIL analyses if the chromosome II QTL represented a polymorphism in a gene specifically involved in the response to daumone. Indeed, this would be consistent with the suggestion that no candidate dauer pheromone receptors have been isolated because different receptors respond to the different active compounds in the pheromone [15]. Hence, the disruption of the receptor for one component of dauer pheromone would not be expected to produce a dauer defective phenotype. Within this region there are many predicted 7-transmembrane receptors [11,23], and such receptors have been implicated in the response to dauer pheromone by the finding that the G-protein α-subunits GPA-1 and -2 are involved in the response to pheromone [30]. One possibility is that one of these genes is the basis of the QTL.

This work has shown that the larval dauer/non-dauer developmental decision and adult reproduction are affected by environmental conditions and that this varies among *C. elegans *RILs. These analyses have been undertaken on natural variation in these traits, thereby reflecting some aspect of how these traits have evolved and, or are maintained. These results further suggest the hypothesis that the same, or closely linked genes, may co-ordinate the response of these different life-history traits to environmental stress and that there are different evolved strategies by which these traits can be deployed. Further, detailed genetic analyses are required to fully resolve this.

## Conclusion

1. *C. elegans *alters both its propensity to form dauer larvae and its adult rate of reproduction and lifetime fecundity, in response to environmental stress.

2. *C. elegans *isolates vary in their dauer larva formation and adult reproductive phenotypes and how these vary in response to stress. Changes in dauer larva development and adult reproductive strategy are related, shown by the correlation of these traits among RILs.

3. QTL analyses have identified regions on chromosome II and X that control dauer larva development and population size. The chromosome II QTL was further resolved to genetically separate the control of these two phenotypes.

4. Genetic analysis of the chromosome II QTL affecting dauer larva formation, resolved this to a *c*. 210 Kbp region containing no genes known to be involved in dauer development.

5. *C. elegans *has a co-ordinated larval and adult response to environmental stress controlled, at least in part, by physically close genomic regions.

## Methods

### Worms

*C. elegans *isolates N2 and DR1350 were used for the construction of recombinant inbred lines (RILs) and nearly isogenic lines (NILs). All *C. elegans *lines were maintained on standard NGM plates with an *Escherichia coli *OP50 food source [31]. Strains were obtained from the *Caenorhabditis *Genetics Center. RILs were generated as previously described [19]. Briefly, this involved maintaining N2 × DR1350 F1 cross progeny, produced from both N2 hermaphrodite by DR1350 male crosses and reciprocal matings, individually and allowing them to self-fertilise for at least 30 generations, at which point they were cryopreserved. In total 200 RILs were produced. NILs were produced by backcrossing N2 × DR1350 F1 cross-progeny to N2 for nine generations and using polymorphic markers (see below) to genotype the progeny at every generation to identify lines with the desired genotype. All resultant NILs were also genotyped for six markers on other chromosomes to confirm homozygosity for N2 alleles.

### Dauer larva formation assays

Dauer pheromone extract was prepared from N2 liquid culture media as previously described [32]. A single batch of pheromone was used for all assays of the RILs. This batch of pheromone was different to that used previously [19] and so the quantities reported here are not comparable to this previous report [19]. Artificial dauer pheromone, so-called daumone, was synthesized according to the published method [17]. This artificial pheromone, daumone, was used for all assays of the NILs. Subsequently, it has been reported that daumone is one of three molecules that comprise *C. elegans *dauer pheromone and that daumone induces the lowest level of dauer larva formation, compared with the other components of dauer pheromone [18]. All dauer formation assays were carried out as previously described [19], with the quantities of pheromone and, or food varied as described below. All dauer assays were performed at 25°C with worms maintained on 3.5 cm diameter plates containing 2 mL of dauer larva formation assay agar [19].

For the RILs, the dauer larva formation phenotype was measured at both high and low food concentrations against a standard concentration of dauer pheromone (15 μL/mL agar). To prepare the high and low food concentrations, liquid cultures of *E. coli *OP50 were grown overnight to saturation, centrifuged and the media removed, and resuspended, in water, at 2 and 1% w/v, respectively. RILs were assayed in 10 separate assays, with N2 and DR1350 controls included in each assay. Within each assay, there were three or four replicate plates for each RIL, and between five and ten replicate plates for N2 and DR1350 each at both food concentrations. Plates on which there were fewer than 20 worms and plates on which larvae could not be classified as dauer or non-dauer larvae with confidence, were excluded from all further analyses. Further, when data were only available for a single plate at one combination of conditions, these data were also excluded. The dauer formation phenotype of the NILs was measured at low food concentrations as described above.

### Population growth

To measure the population growth of 48 randomly selected RILs, L1 stages were obtained by allowing eggs liberated from hypochlorite treated gravid hermaphrodites [31], to develop on NGM plates in the absence of food for 24 hours at 19°C. These arrested L1s were then individually placed on fresh 5.5 cm diameter NGM plates seeded with 100 μL of a stock OP50 food source and maintained at 19°C. The population that grew from these individual L1s were assayed 5, 7 or 9 days later. This was done on day 5 by directly counting the worms (all larval stages and adults) visible on each plate; on days 7 and 9, worms were washed from the plates with M9 buffer, and the number of worms (all larval stages and adults) determined by dilution. Plates on which the worms failed to grow and plates on which the worms had burrowed into the agar were excluded from the analysis. In this manner, the population growth of 48 RILs was measured in 8 experimental blocks, each of which contained 15 replicate plates for 6 RILs. The parental lines, N2 and DR1350, were also included within each experimental block.

### Fecundity assays

The lifetime fecundity and the rate of reproduction, *q *(see Statistical analyses, below), were determined for 12 RILs chosen to be representative of the range of dauer development phenotypes observed among the 48 RILs whose population growth had been determined. Late-stage L4s were transferred individually to NGM plates that had been previously seeded with an excess of OP50 food source, and the worms were then transferred to fresh plates every 8 hours, until egg laying had ceased. The plates from which the hermaphrodites had been removed were then maintained for 24 hours to allow the eggs to hatch and larvae to develop, which were then counted. This was done in two experimental blocks, with the parental lines, N2 and DR1350, included in each block, with 15 replicate plates for each for line at 19°C.

To determine the effect of different concentrations of food on lifetime fecundity and reproductive timing, these analyses were repeated except that the NGM plates were seeded with a food source consisting of 100 μL of 100, 50, 25, 12.5 or 6.25% w/v OP50 diluted in LB medium. Prior to the addition of these food sources, all plates were supplemented with streptomycin to a final concentration of 50 mg/mL, to prevent the growth of OP50. At low food concentrations, worms leave the site of the food and can be lost from an experiment; to prevent this, heated, sterile, 1 cm diameter brass rings were individually heated and melted into the agar prior to the application of the food and the worms, which were placed, and contained, within the rings [33]. This was done for 6 RILs, which were representative of the range of dauer development phenotypes observed among the 12 RILs analysed above and the parental lines, N2 and DR1350, with 25 replicate plates for each line at each food concentration at 19°C. Plates on which worms had desiccated on the walls of the brass rings or in which worms had burrowed into the agar, were excluded from the analysis.

The lifetime fecundity and reproductive timing of five *eat *mutants (DA606 *eat-10(ad606)*, DA522 *eat-13(ad522)*, DA573 *eat-14(ad573)*, DA602 *eat-15(ad602)*) [21] and N2 were determined, as described above, but without the use of brass rings, for individual worms with a food source consisting of 100 μL of 100, 50, 25 or 12.5% w/v OP50 diluted in LB medium. At 100% w/v OP50, 25 replicate plates of each strain were used; 60 replicate plates were used for 50 and 25% w/v OP50 and 70 replicate plates used for 12.5% w/v OP50 at 19°C.

### Statistical analyses

Dauer development and lifetime fecundity were analysed using generalized linear models (GLMs), with LINE and, as appropriate, FOOD and, or PHEROMONE concentration, ASSAY and TIME fitted as factors. When included in analyses, ASSAY was fitted such that LINE was nested within ASSAY. All analyses were performed in Minitab and all proportion data were arcsine transformed prior to analysis. All data presented in the text, figures and tables has been back transformed.

To determine the rate of reproduction, *q*, the daily cumulative fecundity, expressed as a proportion of lifetime fecundity, was determined for each worm. This gives a relationship where the proportion of eggs laid increases from 0 on day 0 and converges to an asymptote of 1 on the final day of egg laying. The reproductive timing, expressed as the cumulative proportion of eggs laid, can then be described by y = 1 - e^(-*qx*)^, where *q *is the rate of reproduction and *x *is the day of egg laying. The reproductive rate, *q*, was then estimated individually for each worm by iteratively determining the value that gave the best fit to the data, which was determined by maximum likelihood using negative log likelihoods. A large value of *q *means that a greater proportion of eggs are laid earlier compared with a low value of *q*. These rates of reproduction, *q*, of different lines and under different conditions were analysed by ANOVA.

The lifetime fecundity and reproductive timing of individual worms were also used to calculate the expected population size after 5, 7 or 9 days and these predicted values were correlated to the observed population sizes. Expected population size was calculated using a model in which the lifetime fecundity and reproductive schedule were used to calculate the number of eggs laid in a particular 8 hour period (see additional file 3). The delay between an egg being laid and it, in turn, reproducing was then estimated using data on the timing of embryogenesis and the time from hatch to first egg laying. This process was iterated over the desired period of time, to determine the total population size.

### QTL analyses

Candidate polymorphic markers that would differentiate N2 and DR1350 were screened by RFLP or direct sequence analysis. Three groups of candidate markers were screened: single nucleotide polymorphisms (SNPs) that differentiated N2 and DR1350 [22]; SNPs that differentiate N2 and CB4856 [23,34] and a number of intergenic and intronic regions that were within regions not covered by the two other groups of candidate markers (see additional file 1 for all regions screened). RILs were genotyped either by RFLP analysis or by allele-specific PCR.

Marker order was based on physical position in the N2 genome [23]. Genetic distance between neighbouring markers was calculated from the proportion of recombinants between the markers in the RILs using Kosambi's mapping function [35]. Segregation distortion from an expected 1:1 DR1350: N2 ratio was tested by χ^2 ^analysis for each marker with Bonferroni-corrected significance levels.

QTL mapping was performed using QTL cartographer [36]. In total, nine traits from three experiments were analysed. Dauer development in response to different food concentrations, from which dauer larva formation at high (2% w/v) and at low (1% w/v) food concentrations, and the plasticity of dauer larvae formation, which was calculated as the difference in the proportion of dauer larvae formed between low and high food concentrations, were analysed. Dauer development in response to different pheromone concentrations from previously available data [19], from which a directly analogous set of traits concerning dauer development in response to different pheromone concentrations were analysed. The population size at day 5, 7 and 9 was also analysed. Each trait was initially investigated by single marker analysis. Data were then analysed by composite interval mapping (CIM), using model 6, forward and backward stepwise regression, a 10 cM window size and a 2 cM walking speed. Genome-wide significance levels, *p *< 0.05, for each trait were determined by performing 1000 permutations on the data. Effects size and R^2 ^values for significant QTLs were determined from these CIM analyses. To better control for the analysis of multiple traits, each of the three experiments, were also analysed by multiple trait CIM (MT-CIM) [36,37]. Analysis settings were as described above; genome-wide significance levels, *p *< 0.05, were determined by performing 1000 permutations on the data. GxE interactions were investigated in two ways. Firstly, the plasticity of dauer larvae formation, calculated as described above, was analysed by CIM as a separate trait. Secondly, GxE interactions were analysed as part of the multiple trait CIM analyses. Epistasis between identified QTLs was assessed by multiple interval mapping (MIM).

## Authors' contributions

MEV conceived and directed the study. SCH devised and executed the dauer larva phenotyping, genotyping, all molecular genetic and genetic analyses and data analyses; AS undertook the population growth and fecundity assays. The manuscript was written by SCH and MEV. All authors read and approved the final manuscript.

## Supplementary Material

Additional file 1 **Genomic regions and markers screened in DR1350**. (A) Genomic regions sequenced in DR1350, showing the physical location, the size of the amplified region and the PCR oligonucleotide primers (5'-3') and, where appropriate, the candidate SNP contained within the amplified region [23]. (B) N2/CB4856 RFLP polymorphisms screened in DR1350, showing the polymorphic marker [23], the chromosome and physical location (Mbp), the restriction endonuclease used and the PCR oligonucleotide primers (5'-3'). No N2/DR1350 polymorphisms were detected. Click here for file

Additional file 2 **Candidate genes underlying the chromosome II QTLs**. Genes and pseudogenes predicted to occur in N2 within the defined regions of the chromosome II QTLs for (A) dauer larva development and (B) for population size on day 5. For each gene, the Wormbase ID number, gene name, gene description and position and, where available, any RNAi phenotypes, the expression pattern and gene ontology (GO) terms are given [23]. 7TM, 7-transmembrane; Adl, adult lethal; Bli, blistered, Bmb, organism morphology abnormal; Ced, cell death abnormal; Clr, clear; Dpy, dumpy; Egl, egg laying abnormal; Emb, embryonic lethal; Gro, slow growth; Let, lethal; Lva, larval arrest; Lvl, larval lethal; Muv, multivulva; Pch, patchy colouration; Prl, paralysed; Pvl, protruding vulva, Ric, aldicarb resistant; Rup, exploded through vulva; Sck, sick; Sma, small; Ste, maternal sterile; Stp, sterile progeny; Unc, locomotion abnormal. Click here for file

Additional file 3 **Model used in the calculation of predicted population size**. The equation used in the calculation of the predicted population size and the definition of the parameters. Click here for file
