# Quantitative genetic analysis of life-history traits of *Caenorhabditis elegans *in stressful environments

**DOI:** 10.1186/1471-2148-9-96

**Published:** 2009-05-12

**Authors:** Simon C Harvey, Alison Shorto, Mark E Viney

**Affiliations:** 1 School of Biological Sciences, University of Bristol, Woodland Road, Bristol, BS8 1UG, UK

## Abstract

In Figure 1 of [Harvey et al (Evolutionary Biology 2008, 8:15)] the plotted data were inverted. The correct Figure is shown below. The text and statistical analyses in [Harvey et al (Evolutionary Biology 2008, 8:15)] are correct.

## Correction

In Figure 1 of [1] the plotted data were inverted. The correct Figure is shown below. The text and statistical analyses in [1] are correct.

**Figure 1:**
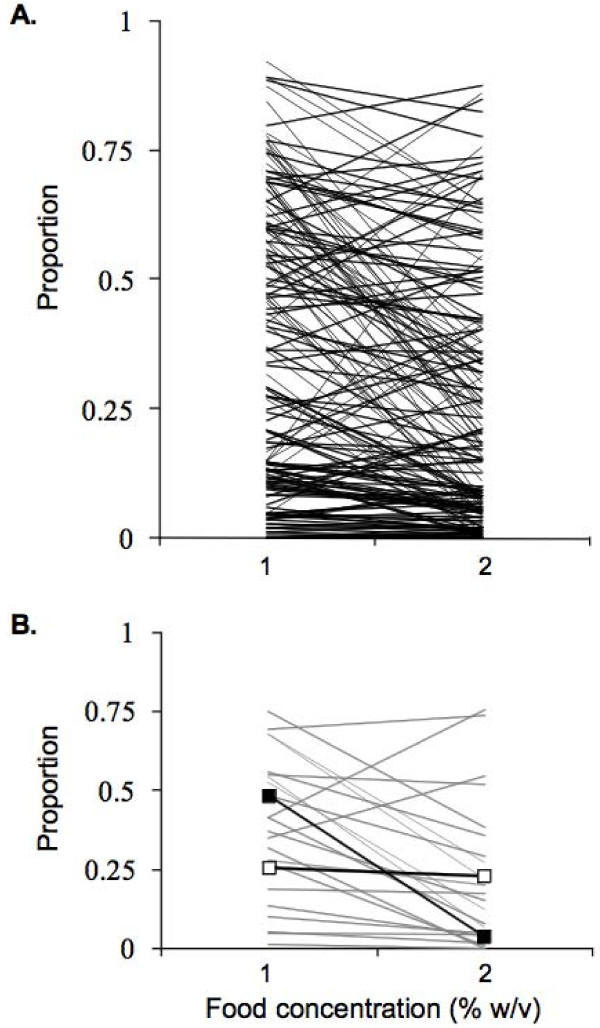
***C. elegans *varies in its dauer larvae formation phenotype**. (A) The proportion of dauer larvae that developed at two concentrations of food (1 and 2% w/v *E. coli *OP50 in water) for 163 RILs and (B) this for 21 RILs and N2 (―■―) and DR1350 (―□―) in one representative assay.
