# Supplementary material for: Quantitative genetic analysis of life-history traits of Caenorhabditis elegans in stressful environments
Source: BMC Evol Biol. 2008 Jan 22;8:15. doi: 10.1186/1471-2148-8-15 (PMC2267162; doi:10.1186/1471-2148-8-15)
Supplement: Additional file 3 — Model used in the calculation of predicted population size. The equation used in the calculation of the predicted population size and the definition of the parameters. [file 1471-2148-8-15-S3.DOC]

### Additional file 3 – Model used in the calculation of predicted population size

In order to determine whether lifetime fecundity and reproductive schedule were sufficient to predict population size, a model was produced in which lifetime fecundity and reproductive schedule were used to predict the maximum population size at different times. This model was adapted to use reproductive schedule data from 8 hour periods rather than from days. This was done by dividing each day into three 8 hour time periods.

Equation 1

$$\sum_{d=1}^{d-1} \sum_{k=5}^{kf} Ek*n(k,d)$$

List of symbols used in Equation 1 and their definitions

| **d** | **Day of observation** |
| --- | --- |
| d-1 | Day before the day of observation. The equation gives the number of eggs and worms present on any particular day. Because eggs take a day to hatch, the number of worms present on any particular day is the number of eggs and worms present on the previous day. |
| k | Age of worm. This includes the time taken for the egg to hatch, which is one day, and development from L1 to adult, which is three days. Therefore at k=1 an egg is laid; at k=5 reproduction starts. |
| kf | Age at which a worm ceases egg laying. |
| Ek | Number of eggs produced by a worm at age k |
| n(k, d) | Number of worms of age (k) on day (d) |
